# Prognostic and Predictive Value of KRAS Mutations in Advanced Non-Small Cell Lung Cancer

**DOI:** 10.1371/journal.pone.0064816

**Published:** 2013-05-28

**Authors:** Jong-Mu Sun, Deok Won Hwang, Jin Seok Ahn, Myung-Ju Ahn, Keunchil Park

**Affiliations:** Division of Hematology-Oncology, Department of Medicine, Samsung Medical Center, Sungkyunkwan University School of Medicine, Seoul, Korea; The University of Texas M. D. Anderson Cancer Center, United States of America

## Abstract

Clinical implications of KRAS mutations in advanced non-small cell lung cancer remain unclear. We retrospectively evaluated the prognostic and predictive value of KRAS mutations in patients with advanced NSCLC. Among 484 patients with available results for both KRAS and EGFR mutations, 39 (8%) had KRAS and 182 (38%) EGFR mutations, with two cases having both mutations. The median overall survivals for patients with KRAS mutations, EGFR mutations, or both wild types were 7.7, 38.0, and 15.0 months, respectively (P<0.001). The KRAS mutation was an independent poor prognostic factor in the multivariate analysis (hazard ratio = 2.6, 95% CI: 1.8–3.7). Response rates and progression-free survival (PFS) for the pemetrexed-based regimen in the KRAS mutation group were 14% and 2.1 months, inferior to those (28% and 3.9 months) in the KRAS wild type group. KRAS mutation tended to be associated with inferior treatment outcomes after gemcitabine-based chemotherapy, while there was no difference regarding taxane-based regimen. Although the clinical outcomes to EGFR tyrosine kinase inhibitors (TKIs) seemed to be better in patients with KRAS wild type than those with KRAS mutations, there was no statistical difference in response rates and PFS according to KRAS mutation status when EGFR mutation status was considered. Two patients with both KRAS and EGFR mutations showed partial response to EGFR TKIs. Although G12D mutation appeared more frequently in never smokers, there was no difference in clinical outcomes according to KRAS genotypes. These results suggested KRAS mutations have an independent prognostic value but a limited predictive role for EGFR TKIs or cytotoxic chemotherapy in advanced NSCLC.

## Introduction

Non-small cell lung cancer (NSCLC) is a leading cause of cancer-related mortality despite intensive anticancer treatment and improvement of clinical modalities seen in the recent decades. In order to provide more individualized therapy for NSCLC, a great effort has been made, targeting against several signaling pathways which include epidermal growth factor receptor (EGFR). The clinical activity of EGFR targeting agent, tyrosine kinase inhibitor (TKI) such as gefitinib and erlotinib, is closely associated with EGFR mutation status in NSCLC, and the clinical relevance of EGFR mutations as a positive predictive factor for EGFR TKI therapy has been well documented [1]–[5].

Like EGFR mutations, KRAS mutations are frequently appearing genetic changes in NSCLC, found in 15% to 30% of NSCLC among Western patients, although the frequency is lower in Asian patients [6]–[11]. However, the clinical implications of KRAS mutations remain unclear. Although some studies previously identified the KRAS mutation as a poor prognostic factor in NSCLC [12]–[14], others have failed to reproduce those results [6], [8], [15]–[17]. In addition, KRAS mutation has been proposed as a mechanism of primary resistance to EGFR TKI [18], and many studies demonstrated poor clinical outcomes to EGFR TKIs in patients with NSCLC harboring KRAS mutation [7], [9], [19], [20]. However, the analysis of the predictive role of KRAS mutation for EGFR TKI therapy can be confounded by EGFR mutation status [21]. Therefore, the predictive value of KRAS mutation for EGFR TKI therapy should be analyzed with EGFR mutation status being considered.

The inconsistent results regarding the prognostic and predictive values of KRAS mutations are in part caused by the heterogeneity and the small size of study population. In addition, many previous studies were performed in the patients with completely resected lung cancer, making it difficult to find small, but significant impacts of a biomarker on survival or treatment outcomes after chemotherapy.

Interestingly, KRAS mutations were recently suggested as sensitizing tumors to pemetrexed, possibly by upregulation of a microRNA that can downregulate KRAS [22]. This observation may be significant since, if it is true, it could affect how patients are selected in the clinical trials investigating new targeting agents for the KRAS pathway as well as how patients are treated in clinical practice.

The purpose of this study was to evaluate whether there is any difference in the treatment outcomes to various types of chemotherapeutic regimens according to the KRAS mutation status and also to investigate the prognostic role of this biomarker.

## Patients and Methods

### Patients and data collection

The study population included patients who were histologically diagnosed of advanced NSCLC at Samsung Medical Center between January 2006 and January 2011. Among them, patients who received palliative chemotherapy and had tumors known for both KRAS and EGFR mutation status were included in this study.

Baseline characteristics and clinical outcomes for the administered chemotherapeutic regimens consisting of first- to third-line chemotherapy were retrospectively reviewed. Smoking status was defined as never (<100 lifetime cigarettes), former (quit ≥1 year before diagnosis), or current smokers (quit <1 year before diagnosis). The amount of smoking was categorized as zero, 30 pack-years or less, and more than 30 pack-years. Chemotherapeutic regimens were categorized into 4 types: pemetrexed-based, gemcitabine-based, taxane-based (paclitaxel or docetaxel) regimens, and EGFR TKI (gefitinib or erlotinib). The response outcome to chemotherapy was defined based on Response Evaluation Criteria Evaluation in Solid Tumors (RECIST). The study was approved by the Institutional Review Board of Samsung Medical Center. The requirement of informed consent was waived as the study was based in the retrospective analyses of existing administrative and clinical data.

### EGFR and KRAS mutation testing

Tumor specimens for all patients in this study were obtained from diagnostic or surgical procedures. The mutational analyses of *EGFR* (exons 18–21) and *KRAS* (exons 2, 3) were performed by directional sequencing of polymerase chain reaction (PCR) fragments amplified with genomic DNA from paraffin-embedded tissue. PCR was performed in a 20 µL volume containing 100 ng of template DNA, 10 x PCR buffer; 0.25 mMdNTPs, 10 pmol primers and 1.25 U Taq DNA polymerase (iNtRON, Korea). PCR products were electrophoresed on 2% agarose gels and were purified with a QIAquick PCR purification kit (QIAGEN, Hilden, Germany). Bidirectional sequencing was performed using the BigDye Terminator v 1.1 kit (Applied Biosystems, Foster City, CA, USA) on an ABI 3130xl genetic analyzer (Applied Biosystems, Foster City, CA, USA).

### Statistical analysis

The relationship between KRAS or EGFR mutations with other clinicopathologic characteristics was analyzed with χ^2^ tests. The logistic regression model was used in the multivariate analysis.

The difference in response rates after each chemotherapeutic regimen according to KRAS or EGFR mutation status was analyzed with χ^2^ tests. Overall survival (OS) and progression-free survival (PFS) were measured from the day of diagnosis of advanced lung cancer and from the start day of each chemotherapeutic regimen, respectively, and were analyzed by Kaplan-Meier estimates and log-rank test. A multivariate analysis was carried out using Cox's regression analysis to assess the independent prognostic role of each clinicopathologic factor.

In addition, OS according to the different types of KRAS mutations were analyzed to determine potential prognostic differences based on KRAS genotypes. Statistical analyses were performed using SPSS 19.0 (SPSS Inc. Chicago, IL), and statistical significance was considered to be P≤0.05.

## Results

### Distributions of KRAS and EGFR mutations

**Figure 1:**
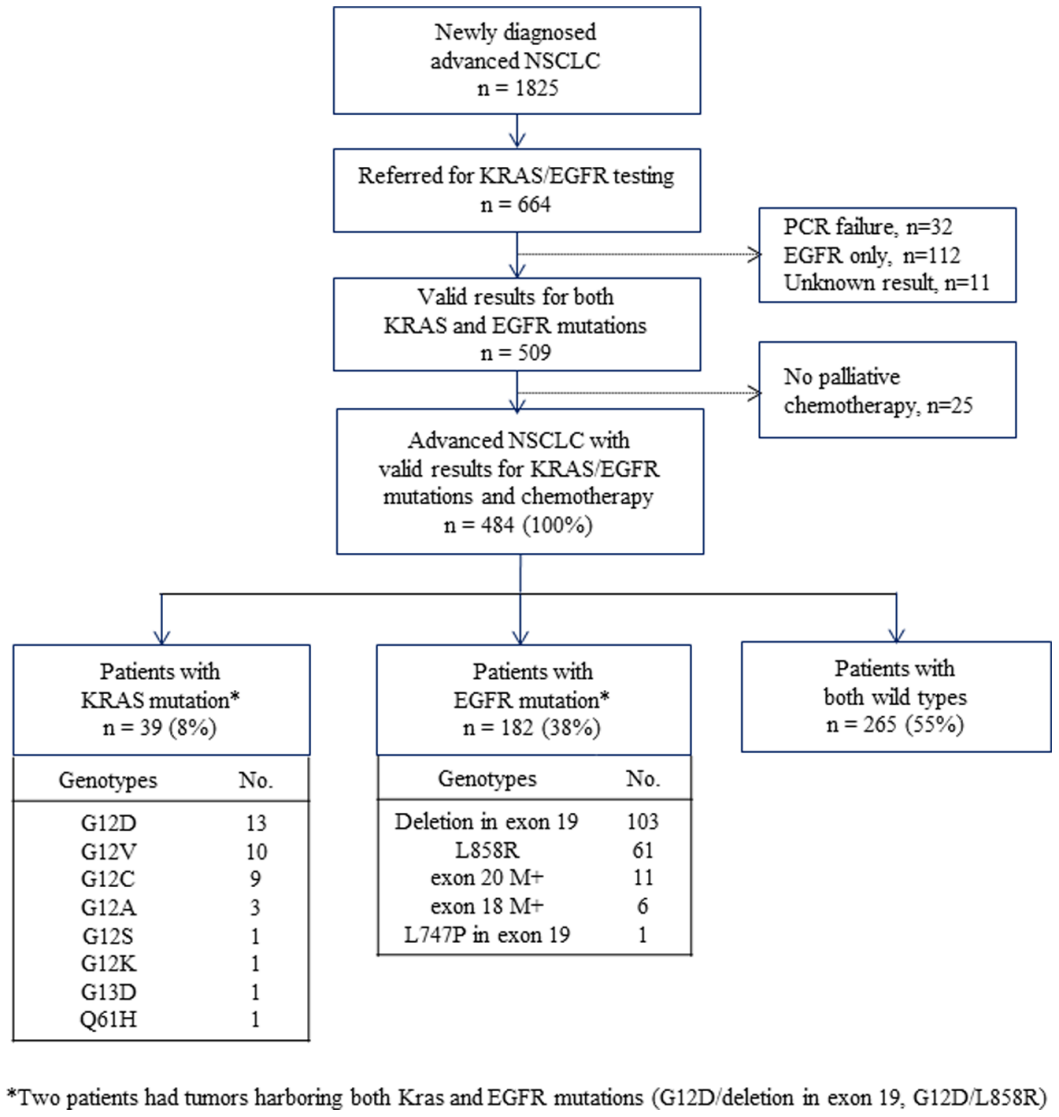
Flow chart of study population. In total, 484 patients with advanced NSCLC with valid results for both KRAS/EGFR mutation status and chemotherapy were included. There were 39 KRAS mutations, 182 EGFR mutations, 265 both wild types. Interestingly, two patients had tumors simultaneously harboring both KRAS and EGFR mutations.

During the study period, 1,824 patients were newly diagnosed of advanced NSCLC at Samsung Medical Center. Among them, 664 (36%) patients were referred for KRAS or EGFR mutation tests. However, only EGFR mutations were tested in 112 patients, neither KRAS nor EGFR mutation test could not be done in 32 patients due to small amount of tumor DNA, and 11 cases who were not tested for both exon 19 and 21 EGFR mutations were classified as unknown mutation status. Consequently, both KRAS and EGFR mutation results were available in 509 patients. We further excluded 25 cases with advanced NSCLC who had not received palliative chemotherapy in our hospital. As a result, 484 patients with advanced NSCLC were included into the analysis (Fig. 1).

KRAS mutations were detected in tumors from 39 (8%) and EGFR in 182 (38%) patients. Two tumors simultaneously had mutations for both KRAS and EGFR (G12V/deletion in exon 19, G12D/L858R). Most KRAS mutations (95%) appeared in codon 12 (13 G12D, 10 G12V, nine G12C, three G12A, one G12S, and one G12K) with one mutation in each codon 13 (G13D) and codon 61 (Q61H). Most EGFR mutations (88%) were typical mutations (103 deletions in exon 19 and 61 L858R mutations in exon 21). The other EGFR mutations were as below: 11 mutations in exon 20 (G796S, S768I, V786M, three duplications, and five insertions), six mutations in exon 18 (three G719A, two S720F, and one G719S), and one L747P mutation in exon 19.

The distribution of KRAS mutations was significantly different by smoking status and the amount of smoking in the univariate analysis (Table 1). In the multivariate analysis, the current smoking status was significantly associated with higher KRAS mutation rates (Odds ratio = 2.7; 95% CI: 1.1–6.7). Although several clinical characteristics such as sex, histology, smoking status, the amount of smoking were associated with EGFR mutation status in the univariate analysis, only adenocarcinoma was found to be an independent predictor for EGFR mutations (Odds ratio = 5.4, 95% CI: 2.1–14.0).

**Table 1:** Demographics of Patients with KRAS or EGFR mutation.

Factors		All cases	KRAS mutation		EGFR mutation	
		No.	No. (%)	P	No. (%)	P
All cases		484	39 (8)		182 (38)	
Sex	Men	279	25 (9)	0.4	76 (27)	<0.001
	Women	205	14 (7)		106 (52)	
Age (years)	<55	180	9 (5)	0.13	75 (42)	0.14
	≥55, <65	157	17 (11)		61 (39)	
	≥65	147	13 (9)		46 (31)	
Histology	Adenocarcinoma	428	39 (9)	0.18	177 (41)	<0.001
	Squamous cell carcinoma	45	1 (2)		5 (11)	
	Others	11	0		0	
Smoking status	Never smoker	232	15 (6)	0.008	117 (50)	<0.001
	Ex-smoker	166	10 (6)		16 (28)	
	Current smoker	86	14 (16)		19 (22)	
Smoking amount (pack-year)	Zero	232	15 (6)	0.01	117 (50)	<0.001
	>0, ≤30	124	6 (5)		40 (32)	
	>30	128	18 (14)		25 (20)	
Clinical stage	IIIB	24	2 (8)	0.96	5 (21)	0.16
	IV	357	28 (8)		134 (38)	
	Postoperative relapse	103	9 (9)		43 (42)	

### Prognostic value of KRAS mutations

With median time to follow-up of 30 months, 312 death events were documented. The OS according to clinical characteristics are shown in table 2. In the univariate analysis, women, younger patients (<65 years), never smokers, and patients with adenocarcinoma, relapsed disease after curative operation, KRAS wild type tumors, and EGFR mutation tumors were associated with longer survival. In the multivariate analysis, KRAS mutations (Hazard ratio [HR] = 2.6, 95% CI: 1.8–3.7) and stage IV (HR = 1.8, 95% CI: 1.1–3.1) were independent predictors of poor prognosis, and EGFR mutations was an independent good prognostic factor (HR = 0.4, 95% CI: 0.3–0.5).

**Table 2:** Overall survival by clinical characteristics.

		Number	Median OS (months)	P (univariate)
Sex	Men	279	15.4	<0.001
	Women	205	29.3	
Age (years)	≥65	147	14.9	<0.001
	<65	337	24.5	
Smoking status	Never	232	27.7	<0.001
	Former	124	15.5	
	Current	128	13.4	
Histology	Adenocarcinoma	428	22.7	<0.001
	Squamous cell carcinoma	45	11.5	
	Others	11	14.4	
Clinical stage	IIIB	24	23	0.002
	IV	357	18.5	
	Postoperative relapse	103	29.5	
KRAS mutation	Yes	39	7.7	<0.001
	No	445	22.6	
EGFR mutations	Yes	182	38	
	No	302	14.2	

Abbreviation, OS: overall survival.

**Figure 2:**
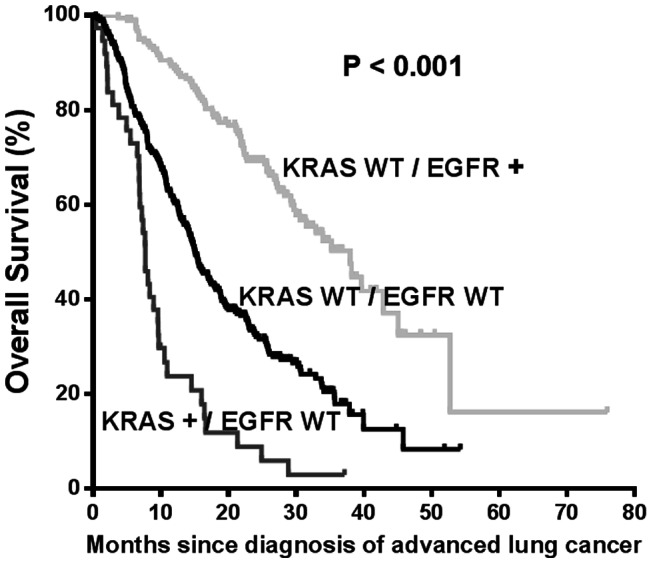
Overall survival by KRAS and EGFR mutation status. The median overall survivals were 38.0, 7.7, and 15.0 months in the groups of EGFR mutation, KRAS mutation, and both wild types, respectively.

To evaluate the relationship between KRAS and EGFR mutations in the survival analysis, patients were divided into three groups: KRAS mutations, EGFR mutations, and both wild type groups. Two patients with both KRAS and EGFR mutations were excluded from this analysis. The survival curves of the three groups were well separated (Fig. 2). The median OS for patients with KRAS mutations, EGFR mutations, or both wild types were 7.7, 38.0, and 15.0 months, respectively (P<0.001). The difference in survival among three groups was also seen among patients with adenocarcinoma with median OS of 7.7, 38.0, and 16.1 months, respectively (P<0.001), on the other hand, it was not significant in patients with squamous cell carcinoma due to small number of patients with KRAS mutation (n = 1) or EGFR mutation (n = 5), (Fig. S1). The statistical significance was also maintained in the comparison of OS between the KRAS mutation and KRAS wild type groups among patients with EGFR wild type tumors (P<0.001).

### Predictive value of KRAS mutations for chemotherapy

In total, 321, 275, 112, and 288 patients received pemetrexed-, gemcitabine-, taxane-based regimens, and EGFR TKI, respectively, as the first-, second-, or third-line chemotherapy. The clinical outcomes after each chemotherapeutic regimen according to KRAS mutation status are shown in table 3. The response rates and PFS for the pemetrexed-based regimen in the KRAS mutation group were 14% and 2.1 months, respectively which were lower than those (28% and 3.9 months) in the KRAS wild type group. Even when the pemetrexed-based regimen was divided into pemetrexed monotherapy and pemetrexed plus platinum chemotherapy, clinical outcomes in the KRAS mutation group were inferior to those in the KRAS wild type group. In particular, there was no response to pemetrexed monotherapy in 13 KRAS mutation tumors. For the gemcitabine-based regimen, patients with KRAS mutation showed lower response rates (18% vs. 36%) and shorter PFS (2.4 vs. 4.2 months) than those with KRAS wild type. For the taxane-based regimen, however there was no difference in treatment outcomes according to KRAS mutation status. Even when the treatment outcomes were analyzed in each line of chemotherapy (the first-, second-, or third-line), their associations with KRAS mutation status showed similar trends.While the response rate to EGFR TKI was 56% in the KRAS wild type group, response in the KRAS mutation group was observed only in two (14%) patients (P = 0.002), both of whom had simultaneous EGFR mutations as well as KRAS mutations. The two patients (one with a G12V/deletion in exon 19 and the other with a G12D/L858R mutation) showed partial response to erlotinib and gefitinib with PFS of 17.7 and 7.1 months, respectively. The PFS for EGFR TKI were 1.6 and 7.0 months in the KRAS mutation and wild type groups, respectively (P = 0.003). When the predictive role of KRAS mutations was analyzed among patients with EGFR wild type tumors, the range of differences in response rates (0% vs. 18%, P = 0.12) and PFS (1.4 vs. 1.8 months, P = 0.09) between the KRAS mutation and wild type groups decreased into the insignificant levels.

**Table 3:** Response Rates and Progression-free Survival by KRAS Mutation Status.

	Total No.		KRAS mutation	KRAS WT	P
Pemetrexed-based regimen	321	No.	28	293	
		Response rate	14%	28%	0.12
		PFS (months)	2.1	3.9	0.002
Pemetrexed monotherapy	166	No.	13	153	
		Response rate	0	19%	0.08
		PFS (months)	1.8	2.6	0.08
Pemetrexed plus platinum	155	No.	15	140	
		Response rate	27%	38%	0.39
		PFS (months)	3.9	4.9	0.004
First-line pemetrexed	152	No.	16	136	
		Response rate	25%	38%	0.3
		PFS (months)	3.2	5.1	0.001
Second-line pemetrexed	107	No.	10	97	
		Response rate	0	25%	0.07
		PFS (months)	1.6	2.6	0.22
Third-line pemetrexed	62	No.	2	60	
		Response rate	0	10%	0.64
		PFS (months)	0.3	2.1	0.16
Gemcitabine-based regimen	275	No.	22	253	
		Response rate	18%	36%	0.09
		PFS (months)	2.4	4.2	0.02
Gemcitabine monotherapy	38	No.	6	32	
		Response rate	0	13%	0.36
		PFS (months)	2.2	2.1	0.44
Gemcitabine plus platinum	237	No.	16	221	
		Response rate	25%	39%	0.25
		PFS (months)	2.4	4.3	0.03
First-line gemcitabine	238	No.	18	220	
		Response rate	22%	40%	0.14
		PFS (months)	2.4	4.4	0.05
Second-line gemcitabine	19	No.	2	17	
		Response rate	0	6%	0.73
		PFS (months)	1.3	2.5	0.22
Third-line gemcitabine	18	No.	2	16	
		Response rate	0	13%	0.6
		PFS (months)	0.7	1.6	0.5
Taxane-based regimen	112	No.	11	101	
		Response rate	27%	27%	0.98
		PFS (months)	1.8	2.5	0.63
Taxane monotherapy	48	No.	5	43	
		Response rate	20%	9%	0.46
		PFS (months)	1.8	1.4	0.83
Taxane plus platinum	64	No.	6	58	
		Response rate	33%	41%	0.7
		PFS (months)	1.4	3.9	0.04
First-line taxane	52	No.	5	47	
		Response rate	40%	49%	0.7
		PFS (months)	2.6	4.3	0.05
Second-line taxane	38	No.	5	33	
		Response rate	20%	12%	0.63
		PFS (months)	1.8	1.5	0.88
Third-line taxane	22	No.	1	21	
		Response rate	0	5%	0.82
		PFS (months)	0.9	1.4	0.35
EGFR TKI	288	No.	14	274	
		Response rate	14%*	56%	0.002
		PFS (months)	1.6	7	0.003
First-line TKI	42	No.	0	42	
		Response rate	N/A	62%	N/A
		PFS (months)	N/A	8.7	N/A
Second-line TKI	188	No.	8	180	
		Response rate	25%*	62%	0.04
		PFS (months)	1.6	9.5	0.1
Third-line TKI	58	No.	6	52	
		Response rate	0%	31%	0.11
		PFS (months)	0.8	1.8	0.06
EGFR TKI in EGFR WT group	125	No.	12	113	
		Response rate	0	18%	0.11
		PFS (months)	1.4	1.8	0.09

Abbreviation, WT: wild type, PFS: progression-free survival, N/A: not applicable.

* Two patients, who showed partial response to TKI in the KRAS mutation group, were also positive for EGFR mutations.

The clinical outcomes after EGFR TKI were strikingly different according to EGFR mutation status: response rates (83% vs. 16%, P<0.001) and PFS (11.8 vs. 1.8 months, P<0.001) in the EGFR mutation versus wild type groups. However, when it was analyzed after EGFR mutations being divided into typical (deletion in exon 19 and L858R) and atypical mutations, only two (G719A, G796S) out of 18 patients with atypical mutations achieved objective response to EGFR TKI.

### Clinical outcomes according to KRAS mutation types

**Figure 3:**
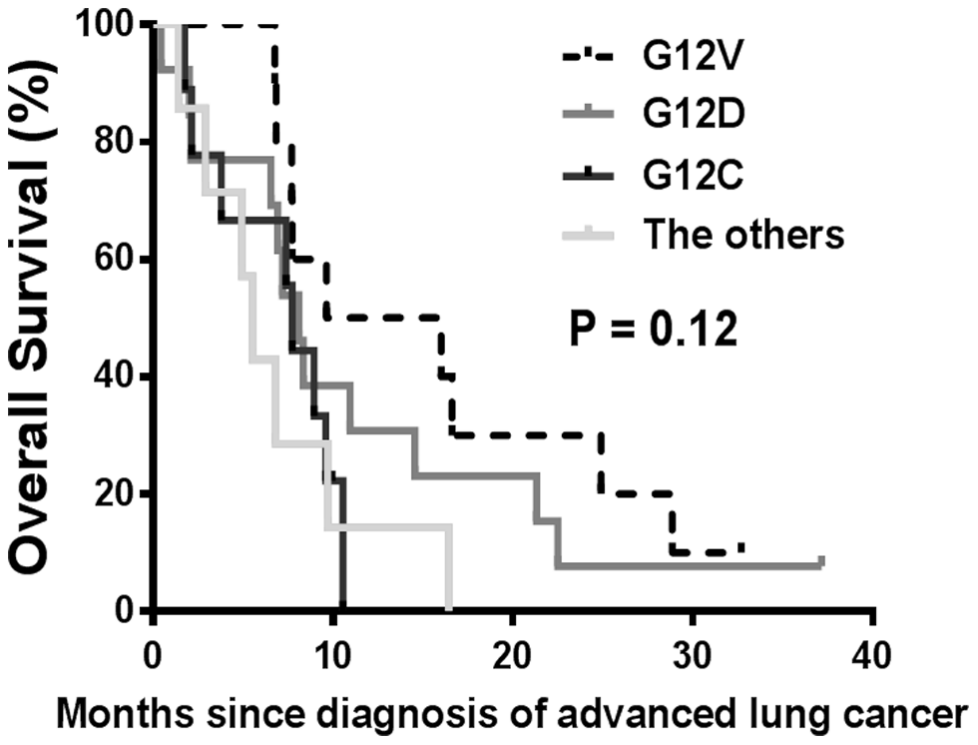
Overall survival according to different types of KRAS mutations. There were ten G12V, 13 G12D, nine G12C, and seven others including three G12A, one G12S, one G12K, one G13D, and one Q61H.

While 62% (8/13) of patients with G12D type were never smokers, only 27% of patients with other KRAS mutation types were never smokers (P = 0.04). When the OS was analyzed after grouping the patients according to four types of KRAS mutations (G12D, G12V, G12C, and the other), the median OS were not different among four groups with the values of 8.1, 9.6, 7.7, and 5.5 months, respectively (P = 0.12), (Fig. 3). In addition, there was no difference in PFS for EGFR TKI therapy according to the KRAS genotypes (P = 0.73). Regarding the response for pemetrexed-based chemotherapy, there was one response (11%, respectively) in each of nine G12V and G12D mutations, zero in seven of G12C, and two responses in the other types.

## Discussion

The present study shows that KRAS mutations are an independent predictor of poor prognosis in patients with advanced NSCLC. However, KRAS mutation status has no role in predicting the clinical outcomes after cytotoxic chemotherapy and is at most a weak predictor regarding EGFR TKI therapy when EGFR mutation status is also considered.

Since KRAS mutation was first found in human lung cancer in 1984 [23], much effort has been expended to evaluate its clinical implication. A meta-analysis identified KRAS mutations as a negative prognostic factor [13]. In addition, it was shown that OS in the KRAS mutation group was significantly poorer than those in the EGFR mutation or in both wild groups with completely resected lung adenocarcinoma [14]. However, a LACE-bio study, which included 1543 patients who had been enrolled into four randomized trials of adjuvant chemotherapy for completely resected NSCLC, recently suggested that KRAS mutations have no prognostic role in patients with completely resected NSCLC [17]. Among many studies with contradictory results, the present study has the merits with a well identified homogeneous study population, analysis controlling for EGFR mutation status, the relatively adequate sample size, and comprehensive analysis on prognostic and predictive values.

While there is no definite relationship between KRAS mutation status and EGFR monoclonal antibody (cetuximab) in advanced NSCLC [10], whether KRAS mutations can predict EGFR TKI response has been contentious. Although many studies noted poorer clinical outcomes after EGFR TKI therapy in the KRAS mutation group compared in the KRAS wild type group [7], [9], [18], [20], that was refuted by findings that KRAS mutation status has no effect on clinical outcomes to EGFR TKI therapy in analysis of patients with EGFR wild types [21]. In our study, although KRAS mutation seemed to predict EGFR TKI responsiveness (KRAS mutation vs. KRAS wild type: 14% vs. 56%, P = 0.002) in the entire study population including patients with or without EGFR mutation, the predicting power of KRAS mutations decreased when EGFR mutation status was also considered. An interesting observation is that there were 20 responses (18%) in 113 patients with both KRAS and EGFR wild type tumors, while there was no response in 12 patients with KRAS mutation and EGFR wild type tumors. The noticeable responses to EGFR TKI in the EGFR wild group could in part be explained by the relatively low sensitivity of direct sequencing EGFR mutation test as noted in a previous study [24]. It suggests that the KRAS mutations have a role as negative selection biomarker for EGFR TKI therapy when the sensitivity of the EGFR mutation test is relatively low.

The irrelevance of KRAS mutations to EGFR TKI responsiveness was directly demonstrated from two cases with both KRAS and EGFR mutations in our study. These two patients achieved durable objective response with EGFR TKIs. The tissues for KRAS and EGFR mutation tests were biopsied at the time of diagnosis; therefore the mutations were de novo in these two patients. Although KRAS and EGFR mutations are known to be mutually exclusive, several episodic cases with both mutations were reported [7], [21], [25]. However, to the best of our knowledge, the clinical outcomes to EGFR TKI in patients with tumors harboring both mutations have been unknown and our study presents the first report of these findings.

In addition to EGFR TKI, the clinical outcomes to other cytotoxic chemotherapeutic regimens could not be predicted by KRAS mutation status. Contrary to the preclinical data reporting that tumors with KRAS mutations, at least the specific genotype such as G12C, would be more sensitive to pemetrexed [22], [26], patients with KRAS mutations showed inferior treatment outcomes to the pemetrexed-based regimen compared with those with KRAS wild types, irrespective of KRAS genotypes. In addition, KRAS mutations tended to be associated with poor treatment outcomes to gemcitabine-based chemotherapy. The overall poor treatment outcomes in the KRAS mutation group to chemotherapeutic regimens seemed to be caused by the negative prognostic value rather than by the negative predictive value of KRAS mutations for specific regimens.

It is hypothesized that different KRAS mutations may have different epidemiologic data, sensitivity to chemotherapy, and survival outcomes. Consistent with a previous report [27], G12D genotype was common in never smokers compared with other genotypes in the present study. Although patients with KRAS genotypes such as G12V or G12C were reported to have poor survival outcome with G12D in previous studies [28], [29], the survival outcomes were not different according to KRAS genotypes in our study. However, the clinical implication of different genotypes is hard to be determined in the present study due to the small sample size.

The present study has several limitations. First, this retrospective study included a cohort who had been tested for KRAS and EGFR mutations in clinical practice which consisted of more women and never smokers. Although this selection bias of the study population could explain the relatively low KRAS mutation rate in our data, the difference from the true value may be small when considering low KRAS mutation rate in Asian population [8]. Secondly, we reviewed chemotherapeutic regimens which were administered as the first-, second-, and third-line therapy. As a result, it was possible that the treatment outcomes after various regimens could be affected by the lines of chemotherapy. However, even when it was analyzed in each line of chemotherapy, the relationship between treatment outcomes and KRAS mutation status was not changed as shown in table 3. Therefore, we do not suspect the heterogeneity of chemotherapy lines had any effects on the analysis of predictive role of KRAS mutations.

This study identified KRAS mutations as an independent prognostic marker but as having a limited role in predicting the treatment outcomes after EGFR TKI or cytotoxic chemotherapy. Based on our observations, patients with KRAS mutant NSCLC could be categorized as a specific group characterized as having poor prognosis without remarkable outcomes to any chemotherapeutic regimen. Although it has not been yet proved clinically, development of agents to inhibit KRAS pathway has been actively pursued [30]–[32]. Further efforts are urgently needed to improve the clinical outcomes of patients with KRAS mutant NSCLC.

## Supporting Information

Figure S1 **Overall survival by KRAS mutation status in patients with adenocarcinoma (a) and squamous cell carcinoma (b).** The difference in survival was significant among patients with adenocarcinoma, on the other hand, it was not significant in patients with squamous cell carcinoma.
(TIF)
